# Validation of a virtual patient and virtual trials method for accurate prediction of tight glycemic control protocol performance

**DOI:** 10.1186/cc9813

**Published:** 2011-03-11

**Authors:** F Suhaimi, AJ Le Compte, S Penning, CG Pretty, JC Preiser, GM Shaw, T Desaive, JG Chase

**Affiliations:** 1 University of Canterbury, Christchurch, New Zealand; 2 University of Liege, Belgium; 3 Erasme University Hospital, Brussels, Belgium; 4 Christchurch Hospital, Christchurch, New Zealand

## Introduction

Effective tight glycemic control (TGC) can improve outcomes, but is difficult to achieve. *In*-*silico *virtual patients and trials offer significant advantages in cost, time and safety for designing effective TGC protocols. However, no such method has been fully validated. This study tests two matched cohorts from the Glucontrol trial treated with different protocols. The goal is to validate the ability of *in-silico *virtual patient models and methods to accurately predict patient-specific and clinical trial glycemic outcomes.

## Methods

The analysis uses records for a 211-patient subset of the Glucontrol trial (Liege, Belgium). Glucontrol-A (*n *= 142) targeted 4.4 to 6.1 mmol/l and Glucontrol-B (*n *= 69) targeted 7.8 to 10.0 mmol/l. Cohorts were matched by APACHE II score, age and sex (*P *> 0.3). The Glucontrol A cohort was slightly older (*P *= 0.04). Virtual patients are created by fitting a clinically validated model to the data, yielding time-varying insulin sensitivity profiles (SI(t)) that create *in-silico *virtual patients. Model fit and intra-patient (forward) prediction are used to validate individual *in-silico *virtual patients. Self-validation (tests A protocol on Group A virtual patients; and B protocol on B virtual patients) and cross-validation (tests A protocol on Group B virtual patients; and B protocol on A virtual patients) assess ability to predict a clinical trial result.

## Results

Model fit errors were small (< 0.25%) for Group A, Group B and the entire cohort (A + B), indicating model fitness. Median prediction errors were 4.3, 2.8 and 3.5% for Group A, Group B and (A + B), indicating individual virtual patients were accurate representations of real patients. Self-validation and cross-validation results were within 1 to 10% of the clinical data for both Group A and Group B. Self-validation indicated clinically insignificant model and compliance errors. Cross-validation clearly showed that the virtual patients enabled by identified patient-specific SI(t) profiles can accurately predict the performance of TGC protocols different from those used to create the virtual patients.

## Conclusions

This study validates these virtual patients and *in-silico *virtual trial methods, and clearly shows they can accurately simulate, in advance, the clinical results of a TGC protocol, enabling rapid *in-silico *protocol design and optimization. It is the first rigorous validation of a virtual *in-silico *patient and virtual trials methodology.

